# Genetic and phenotypic diversity of methicillin-resistant *Staphylococcus aureus* among Japanese inpatients in the early 1980s

**DOI:** 10.1038/s41598-021-84481-6

**Published:** 2021-03-08

**Authors:** Hui Zuo, Yuki Uehara, Yujie Lu, Takashi Sasaki, Keiichi Hiramatsu

**Affiliations:** 1 Department of Microbiology, Juntendo University Faculty of Medicine, 2-1-1 Hongo, Bunkyo-ku, Tokyo, 113-8421 Japan; 2 Department of Clinical Laboratory, St Luke’s International Hospital, Tokyo, Japan; 3 Department of Infectious Diseases, St Luke’s International Hospital, Tokyo, Japan; 4 Center for Infection Control Science Research, Juntendo University Graduate School of Medicine, Tokyo, Japan; 5 Animal Research Center, Sapporo Medical University School of Medicine, Sapporo, Japan

**Keywords:** Infectious diseases, Clinical microbiology

## Abstract

To trace the linkage between Japanese healthcare-associated methicillin-resistant *Staphylococcus aureus* (HA-MRSA) strains in the early 1980s and the 2000s onward, we performed molecular characterizations using mainly whole-genome sequencing. Among the 194 *S. aureus* strains isolated, 20 *mecA*-positive MRSA (10.3%), 8 *mecA*-negative MRSA (4.1%) and 3 *mecA*-positive methicillin-susceptible *S. aureus* (MSSA) (1.5%) strains were identified. The most frequent sequence type (ST) was ST30 (n = 11), followed by ST5 (n = 8), ST81 (n = 4), and ST247 (n = 3). Rates of staphylococcal cassette chromosome *mec* (SCC*mec*) types I, II, and IV composed 65.2%, 13.0%, and 17.4% of isolates, respectively. Notably, 73.3% of SCC*mec* type I strains were susceptible to imipenem unlike SCC*mec* type II strains (0%). ST30-SCC*mec* I (n = 7) and ST5-SCC*mec* I (n = 5) predominated, whereas only two strains exhibited imipenem-resistance and were *tst*-positive ST5-SCC*mec* II, which is the current Japanese HA-MRSA genotype. All ST30 strains shared the common ancestor strain 55/2053, which caused the global pandemic of Panton-Valentine leukocidin-positive MSSA in Europe and the United States in the 1950s. Conspicuously more heterogeneous, the population of HA-MRSA clones observed in the 1980s, including the ST30-SCC*mec* I clone, has shifted to the current homogeneous population of imipenem-resistant ST5-SCC*mec* II clones, probably due to the introduction of new antimicrobials.

## Introduction

*Staphylococcus aureus* (*S. aureus*) is a major opportunistic pathogen that can cause various life-threatening infections, and approximately 20% of healthy human individuals are persistent carriers of this bacterial species^1^. The first case of methicillin-resistant *S. aureus* (MRSA) was identified in the United Kingdom in 1961, only one year after the introduction of methicillin^2,3^. Since then, MRSA has remained a major clinical concern with both hospital-associated and community-associated MRSA (HA-MRSA and CA-MRSA, respectively) infections worldwide^4–6^. The number of deaths related to MRSA infection remains high, and rivals HIV/AIDS infections in its public health impact^7^.

MRSA is generated when methicillin-susceptible *S. aureus* (MSSA) acquires the exogenous *mecA* gene encoding the penicillin-binding protein 2′ (PBP2′), which is carried on a mobile genetic element designated staphylococcal cassette chromosome *mec* (SCC*mec*)^8^. According to the International Working Group on the Classification of Staphylococcal Cassette Chromosome Elements, strains can be classified by SCC*mec* types (I–XIV) based on their combination of *mec* and *ccr* gene complexes^6,9–11^. Each MRSA clone has been categorized by the combination of the chromosomal genotype of the recipient MSSA strain and the genotype of the integrated SCC*mec*. Therefore, multilocus sequence typing (MLST) and SCC*mec* typing by PCR and/or sequencing methods have been widely used as the gold standard methods in molecular epidemiological studies of MRSA^4,12–15^. In addition, whole-genome sequencing (WGS) of bacterial isolates by next-generation sequencing (NGS) technologies has recently become a promising tool for molecular typing, which together with advancing data processing systems, ever-larger NGS datasets and decreased costs has allowed remarkable advances for microbiologists^16^. During the 1970s and 1980s the MRSA epidemic was occurring not only in Japan, but throughout the world^17,18^. However, the molecular typing tools for MRSA isolates were not established until the 2000s. Thus, there have been few reports on molecular epidemiology of MRSA isolates collected before then.

Epidemiological tracking of drug-resistant bacteria using molecular typing tools over time can provide crucial insights into infection control and appropriate use of antimicrobials in clinical practice^19^. Therefore, the aims of this study were to retrospectively review the population structure of Japanese HA-MRSA strains isolated in the early 1980s using MLST, SCC*mec* typing and phylogenetic analysis based on whole-genome single nucleotide polymorphisms (SNPs), and to compare the population structure with that of strains isolated in recent years. Our data shows that in the 1980s, the population structure of Japanese HA-MRSA was remarkably polyclonal, including representative clones that are now rarely found.

## Results

### Prevalence and antimicrobial susceptibilities of MRSA and MSSA isolates in Japanese hospitals during the early 1980s

In total, 194 isolates were identified as *S. aureus*. One hundred and seventy-four patients yielded one strain per patient, whereas ten patients yielded two strains. According to phenotypic and genotypic determination of methicillin-resistance, we identified 20 *mecA*-positive MRSA (10.3%), 8 *mecA*-negative MRSA (4.1%), 3 *mecA*-positive MSSA (1.5%) and 163 *mecA*-negative MSSA (84.0%) strains. Consequently, the phenotypic methicillin-resistant rate of the strains described in this study was 14.4% (28 of 194 strains).

As shown in Table 1, all MRSA strains described in this study were susceptible to levofloxacin, which had not yet entered clinical use in Japan in the 1980s. None of the *S. aureus* strains was resistant to the anti-MRSA agents vancomycin, teicoplanin, linezolid, or arbekacin, irrespective of methicillin resistance. Moreover, 32.1% of MRSA strains (9 of 28 strains) showed resistance to imipenem, which was unavailable in Japan during the period under study, whereas all MSSA strains were susceptible to imipenem.

**Table 1:** Minimum inhibitory concentrations (MICs) of antimicrobial agents against methicillin-resistant and methicillin-susceptible *Staphylococcus aureus* strains.

Antimicrobial agents^b^	MICs (mg/L)							
	MRSA (n = 28)				MSSA (n = 166)			
	Range	MIC^a^_50_	MIC^a^_90_	No. of susceptible strains (%)	Range	MIC_50_	MIC_90_	No. of susceptible strains (%)
Oxacillin	4 to 256	32	128	0 (0.0)	≤ 0.12 to 2	0.25	1	166 (100)
Cefoxitin	4 to 256	16	64	4 (14.3)	≤ 4 to 4	≤ 4	≤ 4	166 (100)
Ampicillin	0.25 to > 16	> 16	> 16	1 (3.6)	≤ 0.12 to > 16	4	16	68 (41.0)
Cefazolin	≤ 0.5 to > 16	> 16	> 16	9 (32.1)	≤ 0.5 to 2	≤ 0.5	1	166 (100)
Cefmetazole	≤ 1 to > 32	16	32	19 (67.9)	≤ 1 to 4	≤ 1	2	166 (100)
Flomoxef^c^	≤ 0.5 to > 16	4	> 16	–	≤ 0.5 to 2	≤ 0.5	≤ 0.5	–
Imipenem	≤ 0.25 to > 8	2	> 8	18 (64.3)	≤ 0.25	≤ 0.25	≤ 0.25	166 (100)
Gentamicin	≤ 0.25 to > 8	> 8	> 8	12 (42.9)	≤ 0.25 to > 8	≤ 0.25	1	153 (92.2)
Arbekacin^c^	≤ 0.25 to 8	1	4	27 (96.4)	≤ 0.25 to 8	0.5	1	165 (99.4)
Minocycline	≤ 2 to > 8	≤ 2	8	24 (85.7)	≤ 2	≤ 2	≤ 2	166 (100)
Erythromycin	≤ 0.12 to > 4	> 4	> 4	11 (39.3)	≤ 0.12 to > 4	0.5	> 4	145 (87.3)
Clindamycin	≤ 0.06 to > 2	0.12	> 2	16 (57.1)	≤ 0.06 to > 2	0.12	0.25	161 (97.0)
Levofloxacin	≤ 0.25 to 1	0.5	0.5	28 (100)	≤ 0.25 to 2	0.5	0.5	165 (99.4)
Vancomycin	≤ 0.5 to 2	1	1	28 (100)	≤ 0.5 to 2	1	1	166 (100)
Teicoplanin	≤ 0.5 to 2	≤ 0.5	1	28 (100)	≤ 0.5 to 2	≤ 0.5	1	166 (100)
Linezolid	0.5 to 2	1	2	28 (100)	0.5 to 4	2	2	166 (100)
Fosfomycin^c^	≤ 32 to > 128	≤ 32	> 128	–	≤ 32 to > 128	≤ 32	≤ 32	–
Sulfamethoxazole/ trimethoprim	≤ 9.5/0.5	≤ 9.5/0.5	≤ 9.5/0.5	28 (100)	≤ 9.5/0.5 to > 38/2	≤ 9.5/0.5	≤ 9.5/0.5	164 (98.8)

^a^MIC_50_/MIC_90_, MIC required to inhibit the growth of 50% or 90% of the strains, respectively.

^b^MICs of oxacillin and cefoxitin were determined by the agar dilution method; all other MICs were determined by the broth microdilution method.

^c^For arbekacin, CLSI breakpoint of gentamicin was used as a substitute; For flomoxef and fosfomycin, no breakpoints were determined by CLSI.

### Detailed genetic characterizations of MRSA and *mecA*-positive MSSA strains

The results of MLST, SCC*mec* typing, *spa*-typing, toxin profiling, and acquired antimicrobial resistance gene profiling are shown in Table 2.

**Table 2:** Genetic characterization of the strains isolated in this study.

Description	Strain ID	Prefecture	Year isolated	MLST^a^	*spa* type	SCC*mec*^a^ type	Toxin gene profile	Antimicrobial resistance gene profile
*mecA*-positive MRSA^a^ (n = 20)	N98	Okinawa	1983	ST5	t001	I	*seb,seg,sei*	*blaZ,mecA,tet(K),aac(6′)-aph(2′')*
	N279	Miyagi	1983	ST5	t001	I	*seb,seg,sei*	*mecA,erm(A),cml,ant(6)-Ia,ant(9)-Ia,aph(3′)-III*
	N283	Miyagi	1983	ST5	t001	I	*seb,seg,sei*	*blaZ,mecA,erm(A),ant(6)-Ia,ant(9)-Ia,aph(3′)-III*
	N366	Nagasaki	1982	ST5	t001	I	*seb,seg,sei*	*blaZ,mecA,aac(6′)-aph(2′'),ant(6)-Ia,aph(3′)-III*
	N345	Okinawa	1983	ST5	t1088	I	*seb,seg,sei*	*blaZ,mecA,erm(A),ant(9)-Ia*
	N106	Ibaraki	1982	ST5	t002	IIa	*tst,sec,seg,sei*	*blaZ,mecA,erm(A),aac(6′)-aph(2′'), aadD,ant(9)-Ia*
	N315	Fukuoka	1982	ST5	t002	IIa	*tst,sec,seg,sei*	*blaZ,mecA,erm(A),aadD,ant(9)-Ia*
	N201	Fukushima	unknown	ST3368	t2588	II	*sed,seg,sei,sej*	*blaZ,mecA,erm(A),aadD,ant(9)-Ia*
	N37	Tokyo	1983	ST12	t213	NT^a^ (*mec* class A)	*sec*	*blaZ,mecA,erm(A),aac(6′)-aph(2′'),aadD,ant(9)-Ia*
	N296	Miyagi	1983	ST30	t021	I	*sea,seg,sei,*φSa2958PVL	*blaZ,mecA,erm(A),erm(B),tet(K),cat(pC233) ,aac(6′)-aph(2′'),ant(6)-Ia,ant(9)-Ia, aph(2′')-Ia,aph(3′)-III*
	N234	Aichi	1982	ST30	t021	I	*sea,seg,sei,φ*Sa2958PVL	*blaZ,mecA,erm(A),erm(B),tet(M),,aac(6′)-aph(2′'),ant(6)-Ia,ant(9)-Ia,aph(3′)-III*
	N237	Aichi	1982	ST30	t021	I	*sea,seg,sei,*φSa2958PVL	*blaZ,mecA,erm(A),erm(B),ant(6)-Ia,ant(9)-Ia,aph(3′)-III*
	N247	Akita	1983	ST30	t021	I	*sea,seg,sei,*φSa2958PVL	*blaZ,mecA,erm(A),erm(B),aac(6′)-aph(2′'),ant(6)-Ia,ant(9)-Ia,aph(3′)-III*
	N267	Fukushima	1982	ST30	t021	I	*sea,seg,sei*	*mecA,erm(A),erm(B),tet(K),cat(pC233),aac(6′)-aph(2′'),ant(9)-Ia,aph(2′')-Ia*
	N28	Hokkaido	1983	ST30	t021	I	*sea,seg,sei*	*blaZ,mecA,erm(A),aac(6′)-aph(2′'),ant(6)-Ia,ant(9)-Ia,aph(3′)-III*
	N153	Osaka	1983	ST30	t021	IVg	*sea,seg,sei,*φSa2958PVL	*blaZ,mecA,erm(B),tet(K),aac(6′)-aph(2′'),ant(6)-Ia,aph(3′)-III*
	N129	Fukuoka	1983	ST81	t127	IVc	*seh*	*blaZ,mecA,aac(6′)-aph(2′')*
	N200	Fukushima	unknown	ST247	t303	I	–	*blaZ,mecA,erm(A),tet(M),,aac(6′)-aph(2′'),ant(6)-Ia,ant(9)-Ia,aph(3′)-III*
	N203	Fukushima	unknown	ST247	t303	I	–	*blaZ,mecA,tet(M),aac(6′)-aph(2′'),ant(6)-Ia,aph(3′)-III*
	N303	Miyagi	1983	ST247	t303	I	–	*mecA,erm(A),tet(M),aac(6′)-aph(2′'),ant(9)-Ia*
*mecA*-negative MRSA (n = 8)	N101y	Toyama	1983	ST5	t179	–	*seg,sei*	–
	N164	Iwate	unknown	ST8	t681	–	–	*blaZ*
	N349	Okinawa	1983	ST25	t258	–	*seg,sei*	*blaZ*
	N86	Kumamoto	1983	ST30	t1504	–	*sea,seg,sei,*φ108PVL	*erm(A),tet(K),,aac(6′)-aph(2′'),ant(9)-Ia*
	N298	Miyagi	1983	ST50	t518	–	*sei*	–
	N89	Kumamoto	1983	ST81	t127	–	*seh*	*aac(6′)-aph(2′')*
	N179	Ibaraki	1983	ST81	t127	–	*sea,seh*	*blaZ*
	N254y	Niigata	1983	ST81	t127	–	*seb,seh*	*blaZ*
*mecA*-positive MSSA^a^ (n = 3)	N240	Aichi	1982	ST30	t021	I	*sea,seg,sei,*φSa2958PVL	*mecA,erm(A),ant(9)-Ia*
	N83	Kumamoto	1983	ST30	t1504	IVd	*sea,seg,sei,*φ108PVL	*blaZ,mecA,erm(A),erm(B),tet(K),aac(6′)-aph(2′'),ant(6)-Ia,ant(9)-Ia,aph(3′)-III*
	N147	Osaka	1982	ST30	t1504	IVd	*sea,seg,sei*	*mecA,erm(A),aac(6′)-aph(2′'),ant(9)-Ia*

^a^*MLST* multilocus sequence typing, *SCCmec* staphylococcal cassette chromosome *mec*, *spa* Staphylococcus protein A gene, *NT* non-typable.

Among the 31 strains that included phenotypically-identified MRSA and *mecA*-positive MSSA, the most frequent sequence type (ST) was ST30 (n = 11, 35.5%), followed by ST5 (n = 8, 25.8%), ST81 (n = 4, 12.9%) and ST247 (n = 3, 9.7%). SCC*mec* types I, II, and IV were found in 15, 3, and 4 of 23 *mecA*-positive strains, respectively. ST30-SCC*mec* I (n = 7) was the most predominant genotype, followed by ST5-SCC*mec* I (n = 5), ST30-SCC*mec* IV (n = 3), ST247-SCC*mec* I (n = 3), and ST5-SCC*mec* II (n = 2). The current predominant HA-MRSA genotype, *tst*-positive ST5-SCC*mec* II, was identified in only two strains, N106 and N315. Eight PVL-positive strains were identified, all of which were ST30.

Among the acquired antimicrobial resistance genes detected in this study, *mecA* was the most frequent (n = 23), followed by *blaZ* (n = 22), *ermA* (n = 19), *ant(9)-Ia* (n = 19), and *aac(6′)-aph(2′’)* (n = 18). All *mecA*-positive strains had aminoglycoside resistance genes, and multiple strains carried genes related to resistance to macrolide (82.6%), tetracycline (39.1%), and phenicol (13.0%).

Overall, a diversity of MRSA isolates representing separate clones were found to be present during the early 1980s, and diverse genotypes were detected even among MRSA strains exhibiting the same ST.

### Antimicrobial susceptibilities of the strains across SCC*mec* types

In order to consider the mechanisms for the shift in population structure of HA-MRSA strains from polyclonal to monoclonal in recent years, we compared the antimicrobial susceptibilities of MRSA strains across SCC*mec* types (Table 3).

**Table 3:** Minimum inhibitory concentrations (MICs) of antimicrobial agents against *mecA*-positive *Staphylcoccus aureus* and *mecA*-negative methicillin-resistant *Staphylococcus aureus* strains in this study by SCC*mec* type.

Antimicrobial agents^b^	MICs (mg/L)															
	SCC*mec* I (n = 15)				SCC*mec* II (n = 3)				SCC*mec* IV (n = 4)				*mecA-*negative MRSA (n = 8)			
	Range	MIC^a^_50_	MIC^a^_90_	No. of susceptible strains (%)	Range	MIC_50_	MIC_90_	No. of susceptible strains (%)	Range	MIC_50_	MIC_90_	No. of susceptible strains (%)	Range	MIC_50_	MIC_90_	No. of susceptible strains (%)
Oxacillin	0.25 to 256	**64**	**256**	1 (6.7)	64	**64**	**64**	0 (0)	0.25 to 256	2	**256**	2 (50.0)	4 to 32	**8**	**32**	0 (0.0)
Cefoxitin	2 to 256	**32**	**256**	1 (6.7)	16 to 32	**32**	**32**	0 (0)	2 to 64	4	**64**	2 (50.0)	4 to 16	4	**16**	4 (50.0)
Ampicillin	≤ 0.12 to > 16	** > 16**	** > 16**	1 (6.7)	> 16	** > 16**	** > 16**	0 (0)	0.25 to > 16	4	** > 16**	1 (25.0)	0.25 to > 16	**16**	** > 16**	1 (12.5)
Cefazolin	≤ 0.5 to > 16	** > 16**	** > 16**	3 (20.0)	> 16	** > 16**	** > 16**	0 (0)	≤ 0.5 to > 16	1	** > 16**	2 (50.0)	≤ 0.5 to > 16	1	4	7 (87.5)
Cefmetazole	≤ 1 to > 32	16	32	11 (73.3)	16 to > 32	32	** > 32**	1 (33.3)	≤ 1 to 32	2	32	3 (75.0)	≤ 1 to 32	2	8	7 (87.5)
Flomoxef^c^	≤ 0.5 to > 16	8	** > 16**	–	16 to > 16	** > 16**	** > 16**	–	≤ 0.5 to > 16	1	** > 16**	–	≤ 0.5 to > 16	≤ 0.5	4	–
Imipenem	≤ 0.25 to > 8	2	** > 8**	11 (73.3)	8 to > 8	** > 8**	** > 8**	0 (0)	≤ 0.25 to > 8	≤ 0.25	** > 8**	3 (75.0)	≤ 0.25 to > 8	≤ 0.25	≤ 0.25	7 (87.5)
Gentamicin	≤ 0.25 to > 8	** > 8**	** > 8**	5 (33.3)	0.5 to > 8	0.5	** > 8**	2 (66.7)	> 8	** > 8**	** > 8**	0 (0)	≤ 0.25 to > 8	≤ 0.25	8	6 (75.0)
Arbekacin^c^	0.5 to 8	1	4	14 (93.3)	0.5	0.5	0.5	3 (100)	0.5 to 4	0.5	4	4 (100)	≤ 0.25 to 4	0.5	1	8 (100)
Minocycline	≤ 2 to > 8	≤ 2	** > 8**	11 (73.3)	≤ 2	≤ 2	≤ 2	3 (100)	≤ 2	≤ 2	≤ 2	4 (100)	≤ 2	≤ 2	≤ 2	8 (100)
Erythromycin	≤ 0.12 to > 4	** > 4**	** > 4**	3 (20.0)	> 4	** > 4**	** > 4**	0 (0)	0.25 to > 4	** > 4**	** > 4**	1 (25.0)	≤ 0.12 to > 4	0.25	0.5	7 (87.5)
Clindamycin	≤ 0.06 to > 2	0.25	** > 2**	9 (60.0)	> 2	** > 2**	** > 2**	0 (0)	0.12 to > 2	0.12	** > 2**	2 (50.0)	≤ 0.06 to > 2	0.12	0.25	7 (87.5)
Levofloxacin	≤ 0.25 to 1	0.5	0.5	15 (100)	0.5	0.5	0.5	3 (100)	≤ 0.25 to 1	≤ 0.25	1	4 (100)	≤ 0.25 to 0.5	0.5	0.5	8 (100)
Vancomycin	≤ 0.5 to 2	1	1	15 (100)	1	1	1	3 (100)	≤ 0.5 to 1	1	1	4 (100)	≤ 0.5 to 1	≤ 0.5	1	8 (100)
Teicoplanin	≤ 0.5 to 2	≤ 0.5	1	15 (100)	≤ 0.5 to 1	≤ 0.5	1	3 (100)	≤ 0.5	≤ 0.5	≤ 0.5	4 (100)	≤ 0.5 to 1	≤ 0.5	1	8 (100)
Linezolid	0.5 to 2	2	2	15 (100)	1 to 2	2	2	3 (100)	1 to 2	1	2	4 (100)	0.5 to 2	1	2	8 (100)
Fosfomycin^c^	≤ 32 to > 128	≤ 32	** > 128**	–	≤ 32	≤ 32	≤ 32	–	≤ 32 to > 128	≤ 32	** > 128**	–	≤ 32	≤ 32	≤ 32	–
Sulfamethoxazole/ trimethoprim	≤ 9.5/0.5	≤ 9.5/0.5	≤ 9.5/0.5	15 (100)	≤ 9.5/0.5	≤ 9.5/0.5	≤ 9.5/0.5	3 (100)	≤ 9.5/0.5	≤ 9.5/0.5	≤ 9.5/0.5	4 (100)	≤ 9.5/0.5	≤ 9.5/0.5	≤ 9.5/0.5	8 (100)

^a^MIC_50_/MIC_90_, MIC required to inhibit the growth of 50% or 90% of the strains, respectively; bold letters mean MIC greater than the breakpoints.

^b^MICs of oxacillin and cefoxitin were determined by the agar dilution method; all other MICs were determined by the broth microdilution method. SCC*mec,* Staphylococcal cassette chromosome *mec.*

^c^For arbekacin, CLSI breakpoint of gentamicin was used as a substitute; For flomoxef and fosfomycin, no breakpoints were determined by CLSI.

Strains that carried SCC*mec* types I and II were highly resistant to β-lactams including oxacillin, but those that carried SCC*mec* type IV were more susceptible to β-lactams despite being *mecA*-positive. Among the 15 strains carrying SCC*mec* type I, the rate of erythromycin resistance was the highest (80.0%), followed by resistance to gentamicin (66.7%), clindamycin (40.0%), and minocycline (26.7%), which were commonly used antimicrobials at that time. The imipenem susceptibility rate in SCC*mec* type I strains was 73.3% (11 of 15 strains) as compared with 0% in SCC*mec* type II strains (0 of 3 strains) (*p* = 0.043).

The *mecA*-negative MRSA strains (n = 8) were more susceptible to β-lactams, and notably, all strains were susceptible to imipenem except the isolate N89. Also, these strains showed lower MICs for aminoglycosides, minocycline, erythromycin and clindamycin, when compared with *mecA*-positive strains.

### Population structure of MRSA and *mecA*-positive MSSA strains in Japan during the early 1980s

**Figure 1:**
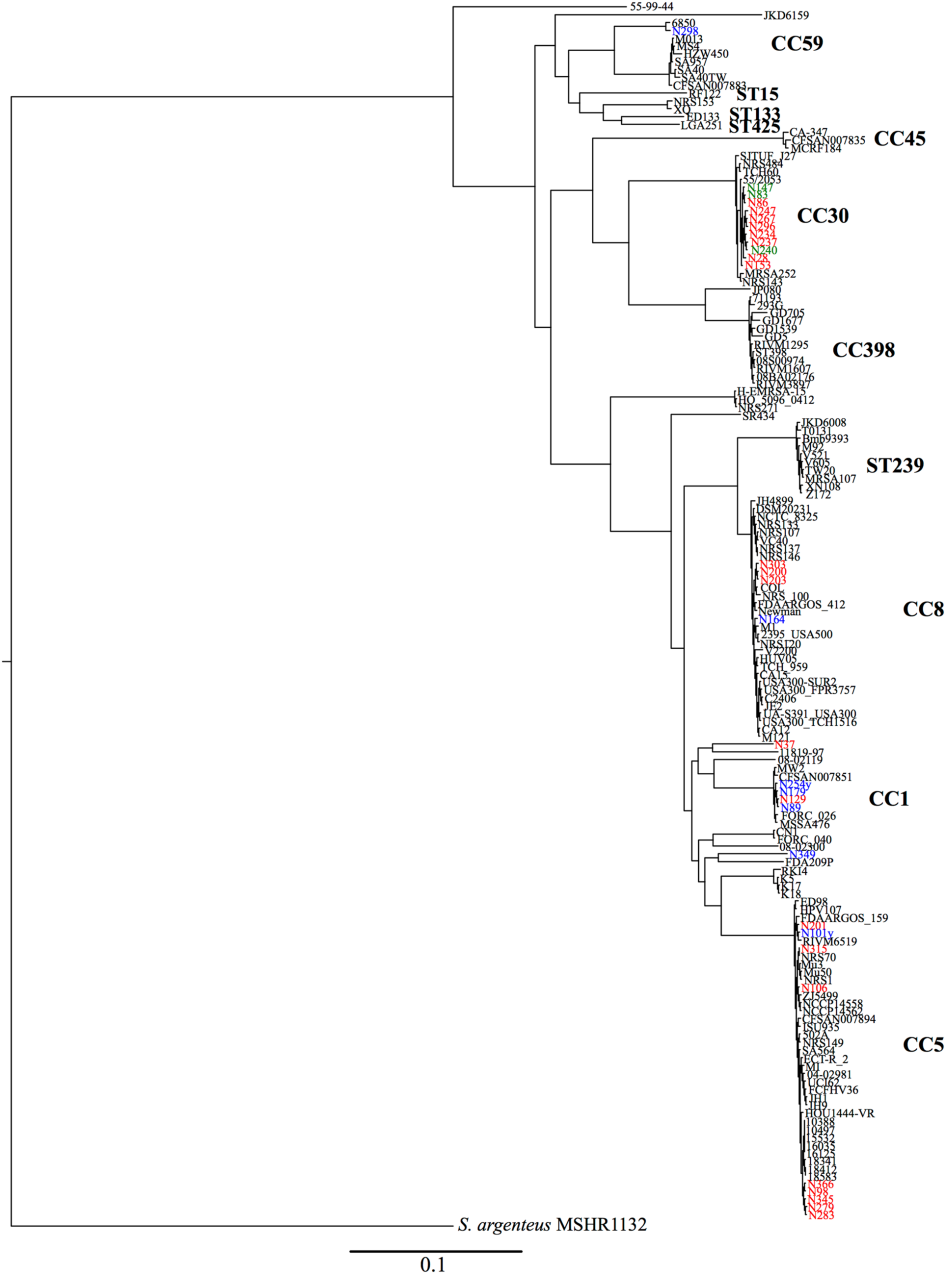
Phylogenetic tree based on whole-genome SNPs in strains in the present study and 125 reference strains. *mecA*-positive MRSA, *mecA*-negative MRSA, *mecA*-positive MSSA isolated in this study and reference strains were indicated in red, blue, green and black letters, respectively. NJ tree was constructed by alignment of 41,910 SNP sites. *S. argenteus* MSHR1132 was used as the outgroup. *CC* clonal complex, *MRSA* methicillin-resistant *Staphylococcus aureus*, *MSSA* methicillin-susceptible *Staphylococcus aureus*, *NJ* neighbor-joining, *SNPs* single nucleotide polymorphisms, *ST* sequence type.

The result of phylogenetic analysis based on whole-genome SNPs of 20 *mecA*-positive MRSA, 8 *mecA*-negative MRSA, 3 *mecA*-positive MSSA, and 125 reference *S. aureus* strains, is shown in Fig. 1.

A neighbor-joining (NJ) tree was constructed by the alignment of 41,910 SNP sites. The percentage of the reference genome (*S. argenteus* MSHR1132) covered by all isolates was 62.95% (1,739,038 of 2,762,785 positions). Strains belonging to the CC30 cluster were most abundant (n = 11), followed by clonal complexes (CC) 5 (n = 9), CC1 (n = 4), and CC8 (n = 4), indicating that the population structure of MRSA strains during the early 1980s was composed of diverse clones.

### Whole-genome SNP analysis of CC30 and CC5 strains

**Figure 2:**
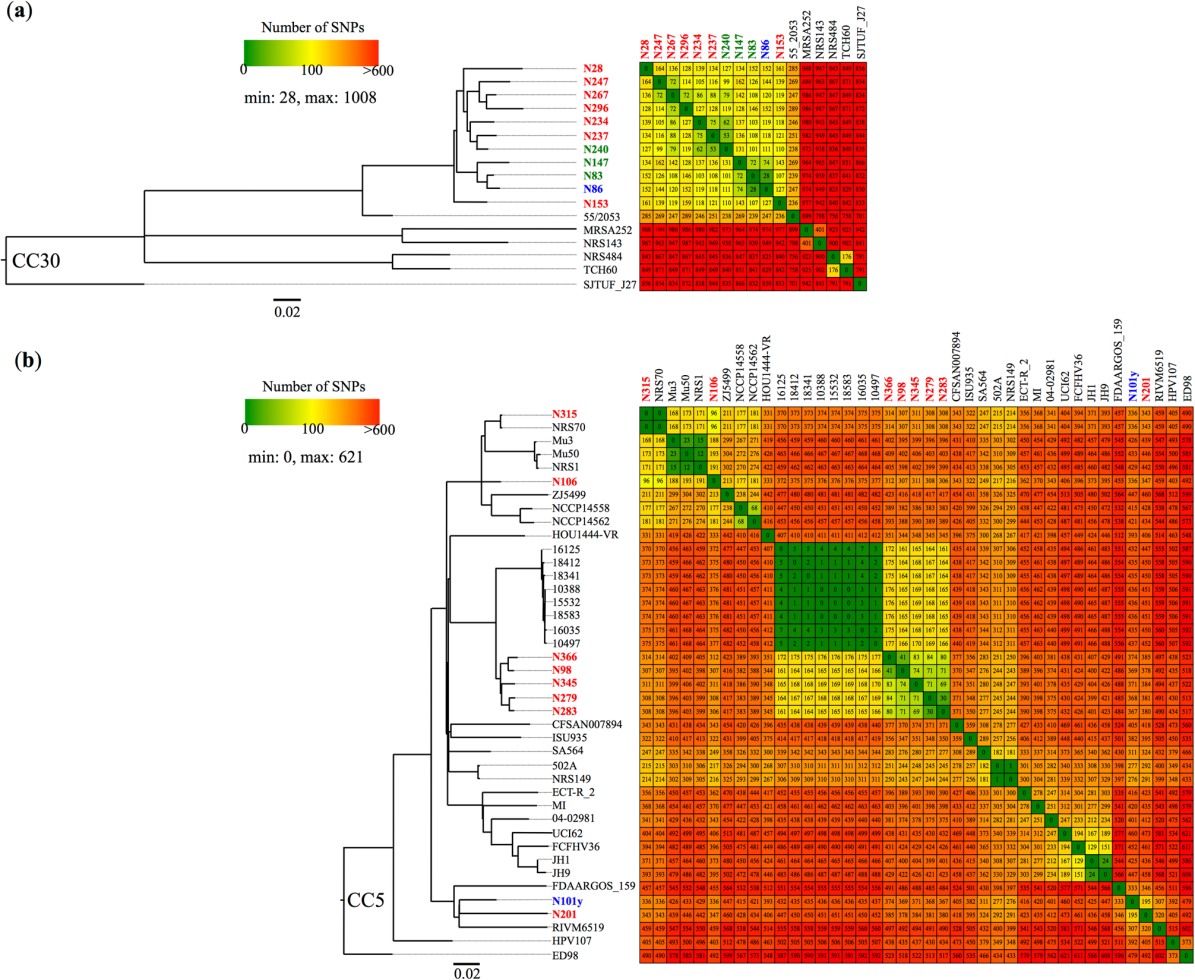
The phylogenetic inter-strain relationships within the same clonal complex based on pairwise SNP differences. (**a**) Phylogenetic tree based on whole-genome SNPs in CC30 strains. *Staphylococcus aureus* strain SJTUF_J27 was used as the outgroup. NJ tree was constructed based on the alignment of 2353 SNP sites. (**b**) Phylogenetic tree based on whole-genome SNPs in CC5 strains. *S*. *aureus* strain ED98 was used as the outgroup. NJ tree was constructed based on the alignment of 3684 SNP sites. *mecA*-positive MRSA, *mecA*-negative MRSA, *mecA*-positive MSSA isolated in this study and reference strains were indicated in red, blue, green and black letters, respectively. The numbers of inter-strain SNP differences were visualized in a red-yellow-green gradient with red indicating the top score (> 600) and green indicating the bottom score (0). *CC* clonal complex, *MRSA* methicillin-resistant *Staphylococcus aureus*, *MSSA* methicillin-susceptible *Staphylococcus aureus*, *NJ* neighbor-joining, *SNPs* single nucleotide polymorphisms.

Detailed pairwise SNPs analysis of 11 ST30 strains described in this study and 6 CC30 reference strains was performed (Fig. 2a). The percentage of the reference genome (SJTUF_J27, ST433) covered by all strains was 89.16% (2,500,756 of 2,804,761 positions) in the SNPs analysis. SNP differences ranged from 28 to 1008. All CC30 strains described in this study clustered into a single clade and were most closely related to the MSSA strain 55/2053 isolated in the United Kingdom in 1955^20^. Strains N83 and N86, which were isolated in Kumamoto in the same year and exhibited the same *spa*-type t1504, showed 28 SNP differences, suggesting a direct horizontal spread within the hospital. However, the SCC*mec* type and antimicrobial resistance gene profiles differed between these two strains, suggesting that these strains were independently acquired by each inpatient from different infectious sources. Similarly, the ST30 strains isolated in this study could be recognized as branches of a clone endemic to Japan, with only small numbers of SNPs ranging from 28 to 164^21,22^.

Next, we performed SNP analysis among 9 CC5 MRSA strains in this study and 32 reference strains (Fig. 2b). The percentage of the reference genome (ED98) covered by all strains was 89.13% (2,517,393 of 2,824,404 positions) in the SNP analysis. SNP differences ranged from 0 to 621. ST5 MRSA strains described in this study clustered into five different clades. Strains N366, N98, N345, N279, and N283 harboring SCC*mec* type I belonged to a single cluster, with the number of SNP differences within the cluster ranging from 30 to 84. Strains N279 and N283, which were isolated in Miyagi in the same year, showed only 30 SNP differences and similar profiles regarding *spa* type, toxin and antimicrobial resistance genes, suggesting transmission events within a short period. Although strains N366 and N98 strains exhibited only 41 SNP differences, these strains were isolated in locations separated by over 750 km of mostly ocean, Okinawa and Nagasaki (Table 2). According to these results, strains exhibiting ST5-SCC*mec* I belonging to this clade could be recognized as part of another endemic clone in Japan, as contrasted with strains 16,125, 18,412, 18,341, 10,388, 15,532, 18,583, 16,035, and 10,497, all ST228 and all isolated at a hospital during the outbreaks in Switzerland^23^, and distinguished from each other pairwise by only 0 to 7 SNPs (Fig. 2b). In contrast, only the strains N315 and N106 exhibiting ST5-SCC*mec* II belong to the same clade with Mu3 and Mu50, which were isolated as HA-MRSA in the 1990s^24^.

These results suggest that MRSA clones exhibiting ST30- and ST5-SCC*mec* I may have already spread as major endemic clones throughout Japan by the early 1980s, whereas ST5-SCC*mec* II had achieved only a minor presence at that time.

## Discussion

Our results suggest that the population structure of Japanese HA-MRSA strains during the early 1980s was notably different from that in recent years. The early 1980s polyclonal structure included ST5- and ST30-SCC*mec* I clones, both of which have become uncommon recently. The recent monoclonal population structure of HA-MRSA strains in Japan, which is composed of imipenem-resistant ST5-SCC*mec* II clone, likely formed over the past several decades, possibly in response to the release of various new antimicrobial agents including imipenem and changes in MRSA treatment strategies from the 1980s onward.

PVL-positive ST30-SCC*mec* I was the most frequent genotype among Japanese HA-MRSA strains in the early 1980s. According to a previous report, nosocomial outbreaks of MRSA exhibiting PVL-positive ST30-SCC*mec* IV occurred frequently in the late 1980s and early 1990s in Japanese hospitals^25^, whereas this genotype comprised only a minor population in the early 1980s as represented in this study. Our results suggest that the population structure of Japanese HA-MRSA strains underwent dynamic replacement through the 1980s. This replacement, largely due to ST30 MRSA clones, has probably resulted from high genetic and phenotypic diversity among ST30 MRSA strains. Indeed, in addition to *mecA*-positive MRSA, ST30 strains rarely isolated today such as *mecA*-negative MRSA and *mecA*-positive MSSA, were also found among the ST30 strains analyzed here. Differences in observed SCC*mec* types and the presence or absence of PVL genes were also noted among these strains.

As previously reported, the CC30 *S. aureus* lineage can be divided into three clusters: Clade 1 (prototype strain 55/2053; PVL-positive and penicillin-resistant MSSA), Clade 2 (prototype strain TCH60; PVL-positive CA-MRSA harboring SCC*mec* type IV), and Clade 3 (prototype strain MRSA252/EMRSA-16; PVL-negative HA-MRSA harboring SCC*mec* type II or IV)^20^. Clade 1 strains cause severe infections and were the epidemic strain type in Europe, the United States, and Australia in the 1950s^26–30^. The percent of *S. aureus* infections caused by Clade 1 strains had dramatically decreased by the mid-1960s, due to methicillin use for the treatment of penicillin-resistant strains^31^. However, according to our results, this clone had re-emerged as HA-MRSA in Japanese hospitals in the early 1980s. Our phylogenetic analysis based on whole-genome SNPs demonstrated that all Japanese ST30 isolates clustered into a single clade including strain 55/2053, suggesting that a Clade 1 strain imported from overseas had acquired SCC*mec* type I, SCC*mec* type IV, or unknown genetic factors and had already undergone diversification in Japanese hospitals by the early 1980s. Consequently, the ST30 strains had likely spread throughout Japan as a nosocomial clone causing a regional outbreak at that time.

This study shows that ST5-SCC*mec* I was the second-most frequent genotype among Japanese HA-MRSA strains in the early 1980s. This genotype is shared by EMRSA-3, which was the most common MRSA clone in the United Kingdom in 1987–1988 along with EMRSA-15 (ST22-SCC*mec* IV) and EMRSA-16 (ST36-SCC*mec* II)^4^. Studies conducted in South America in the late 1990s have identified the Cordobes/Chilean clone, which is genetically related to EMRSA-3 but presents differences in its pulsed-field gel electrophoresis (PFGE) pattern and *spa* type^32–34^. This MRSA clone was also detected at a high rate in hospitals in South Brazil in 2008, suggesting the potential for re-dissemination in Brazil^35,36^. Although this MRSA clone exhibiting ST5-SCC*mec* I has remained uncommon in regions outside of South America in recent years, continuous monitoring is needed to prevent future outbreaks.

Surprisingly, the clones of MRSA resistant to imipenem, such as strain N315, existed before imipenem entered clinical use. We previously reported strain N315, which was imipenem-resistant, *tst*-positive ST5-SCC*mec* II, as a representative strain of the New York/Japan HA-MRSA clone^37^. We reported that strains harboring SCC*mec* type II accounted for a large portion of MRSA in Japanese hospitals in the late 1990s^12,38^. In this study, our results show that a Japanese HA-MRSA lineage exhibiting the same genotype as strain N315 was already circulating as one of the diverse clones in the early 1980s. The phenotypic characteristics of strain N315 was multidrug-resistant, especially to imipenem. In the 1980s, multiple broad-spectrum antimicrobials entered clinical use in Japan, while imipenem/cilastatin was launched in 1987 and was being used as an anti-MRSA agent before the clinical introduction of vancomycin in 1991 in Japan. By contrast with SCC*mec* II strains, strains harboring SCC*mec* type I, which was the predominant genotype in this study and some countries including the United Kingdom in the early 1980s^12,39^, displayed a high rate of imipenem-susceptibility. It was also reported that in vitro exposure to imipenem can select for conversions of heterogeneous-to-homogeneous and Eagle type-to-homogeneous methicillin resistance in *S. aureus* strains via mutations to such chromosomal genes as *vraSR* and *rpoB*^6,13,24,40–43^. Thus, the frequent use of imipenem to treat MRSA infections may have contributed to the selective pressure for imipenem-resistant ST5-SCC*mec* II MRSA between 1980 and 2000, and caused the dynamic population shift in Japanese hospitals from diverse imipenem-susceptible MRSA clones to the monoclonal imipenem-resistant ST5-SCC*mec* II MRSA. The reason why imipenem-resistant clones other than ST5-SCC*mec* II MRSA disappeared in the 1990s is unclear, but some not-yet-understood factors may exist that boost the survival rate of ST5-SCC*mec* II MRSA.

Our results show that the heterogeneous population of diverse clones observed in the 1980s shifted to the homogeneous population of ST5-SCC*mec* II clones from the 2000s onward among Japanese HA-MRSA isolates. However, entering the 2010s, further changes have been occurring in the population structural. It was reported that the population of Japanese HA-MRSA was shifting again in the 2010s from N315-like CC5-SCC*mec* II to CC8- SCC*mec* IV and CC1- SCC*mec* IV, both of which had higher susceptibility to cefotaxime, levofloxacin, clarithromycin and clindamycin^44^. The recovery of antimicrobial susceptibilities, and the history of clonal evolution of HA-MRSA strains from the 1980s to the 2010s, seems to reflect improved recent awareness of appropriate antimicrobial usage.

In this study, multiple MRSA strains exhibiting ST247-SCC*mec* I were isolated in the northeast area of Japan. This genotype is known as the Iberian clone, which was one of the major pandemic MRSA clones until the 2000s^45–48^. Our results show the local existence of the Iberian clone in Japan during the early 1980s. Interestingly, all ST247-SCC*mec* I strains in this study were resistant to imipenem. During the early clinical use of imipenem around the world, the Iberian clone may have undergone spread from the 1990s to the early 2000s. However, the Iberian clone has already been supplanted by the current major epidemic clones^49^.

Intriguingly, multiple *mecA-*negative MRSA and *mecA*-positive MSSA strains, though rarely observed today, were identified in this study, suggesting that methicillin-resistance in *S. aureus* strains of that time had both genetic and phenotypic diversity. These atypical *S. aureus* strains are known as oxacillin-susceptible MRSA (OS-MRSA) or borderline oxacillin-resistant *S. aureus* (BORSA) with oxacillin MICs typically equal to 1–8 μg/mL, which have been reported from various geographic locations for over a decade^50,51^. Although the clinical instances are not frequent compared with typical MRSA, OS-MRSA could have been underestimated because of the discrepancy between the phenotypes and genotypes in clinical laboratories^50^. BORSA can appear as community-acquired infections related to previous antimicrobial drug usage^52^. Our results suggest that OS-MRSA and BORSA strains were already circulating in the early 1980s. All OS-MRSA strains in the present study were ST30 strains isolated in geographically separated regions, and accounted for 30.0% (3 of 10) of *mecA*-positive ST30 strains. Even though *S. aureus* strains with intermediate methicillin-resistance such as OS-MRSA and BORSA were frequently isolated from hospital inpatients in the early 1980s, enhanced selective pressures due to new drug developments may have eliminated them from hospital environments over the past several decades. The intrinsic mechanisms of methicillin-resistance vary from isolate to isolate. The presence or hyper-production of beta-lactamase^53–55^, or the quantity of native PBP proteins and their β-lactam binding affinities^54–62^, or mutations in several chromosomal genes (e.g., *femA*, *femB*, *gdpP*, *yjbH*, and *acrB*) have been presumed to mediate β-lactam resistance^58–62^. Further systematic characterization of these atypical phenotypes will be indispensable in identifying undiscovered genetic traits of methicillin resistance.

In conclusion, this study reveals the alteration in population structure of HA-MRSA strains from the early 1980s onward, probably due to the survival of highly drug-resistant clones that may have arisen in response to new drugs introduced over the past several decades. Our findings clarify the role of diagnostic microbiology for tracking the epidemiology of MRSA, giving important evidence for a close correlation between spread of drug-resistance and appropriate/inappropriate use of antimicrobials. These findings will aid efforts to prevent escalating antimicrobial resistance.

## Methods

### Bacterial strains collection

This study examined a collection of 194 *S. aureus* strains (designated as “N” strains) that were isolated from 184 Japanese inpatients in 22 prefectures between January 1982 and December 1983. A subset of these N strains was previously reported^63^. The isolates were mainly from Fukushima (19, 9.8%), Miyagi (18, 9.3%), Okinawa (18, 9.3%), and Osaka (18, 9.3%) prefectures. Specimen types were as follows: pus (88, 45%), sputum (43, 22%), blood (20, 10%), urine (15, 8%), pharynx (10, 5%), other (7, 4%), and unknown (12, 6%), suggesting that skin and soft tissue infection and respiratory tract infection were major infectious diseases in the study population.

The originally stored isolates were inoculated on BBL Trypticase Soy Agar (TSA) (Beckton Dickinson Japan, Co., Ltd., Tokyo, Japan) and incubated at 37 °C for 24 h. Catalase-positive, Gram-positive cocci that were presumptively identified as staphylococci by colony morphology, were subcultured on TSA. Tube coagulase tests with rabbit plasma (Denka Seiken Co., Ltd., Tokyo, Japan) were performed, and only coagulase-positive staphylococcal strains were selected for further investigation. *S. aureus* was confirmed by a PCR method targeting the thermonuclease (*nuc*) gene after DNA extraction^64^. The *S. aureus* isolates were stored again in sterilized 10% skim milk (Difco Skim Milk, Becton, Dickinson and Co., Franklin Lakes, NJ, USA) at − 80 °C.

### DNA extraction

Chromosomal DNA was extracted from bacterial cultures after single colony isolation on TSA, using the QIAamp DNA Mini Kit (QIAGEN, Hilden, Germany). Purified genomic DNA was used for PCRs and sequencing-based methods.

### Determination of methicillin resistance

We determined phenotypic methicillin resistance in all *S. aureus* strains by evaluating oxacillin and cefoxitin susceptibilities according to Clinical and Laboratory Standards Institute (CLSI) M100-S22 performance standards. In addition, all strains were genetically assessed by two different PCRs for the presence of the *mecA* gene^65,66^. We also confirmed the presence or absence of the *mecA* gene by whole-genome sequencing for phenotypically-identified MRSA strains.

### Antimicrobial susceptibility testing

Minimum inhibitory concentration (MIC) tests for other antimicrobial agents were performed by the broth microdilution method by BBL Mueller–Hinton II Broth (Cation-Adjusted) (CAMHB) (Beckton Dickinson Japan, Co., Ltd., Tokyo, Japan) using the Dry Plate “Eiken” DP32 (Eiken Chemical Co., Tokyo, Japan), containing oxacillin, cefoxitin, ampicillin, cefazolin, cefmetazole, flomoxef, imipenem, gentamicin, arbekacin, minocycline, erythromycin, clindamycin, levofloxacin, vancomycin, teicoplanin, linezolid, fosfomycin and trimethoprim-sulfamethoxazole. For oxacillin and cefoxitin, agar dilution method was also performed using BBL Mueller Hinton II Agar (Beckton Dickinson Japan, Co., Ltd., Tokyo, Japan), with 2% NaCl for oxacillin, in order to measure the detailed MIC values. MICs were examined by visual observation and interpreted according to CLSI M100-S22 performance standards.

The differences in the rates of imipenem susceptibility by SCC*mec* types were evaluated using Fisher's exact test utilizing the fisher. test function in R version 3.5.1 (R Development Core Team). Differences with *p* values < 0.05 were considered significant.

### Molecular typing of MRSA strains

*spa*-typing and multilocus sequence typing (MLST) were carried out as previously reported^67–69^. Direct sequencing of PCR products was performed for *spa* typing and MLST for the *S. aureus* strains. Sequencing reactions were performed using a Big Dye Terminator (version 3.1) Cycle Sequencing Kit with an ABI Prism 3100 genetic analyzer (Applied Biosystems, Thermo Fisher Scientific Inc., Waltham, MA, USA). After assembling both forward and reverse consensus sequences, the *spa* type and MLST were assigned using the RIDOM web server (http://spaserver.ridom.de/) and the PubMLST (https://pubmlst.org/organisms/staphylococcus-aureus), respectively.

SCC*mec* typing (I–V) was performed by a multiplex PCR method reported previously^14^. Thirteen exotoxin genes, encoding staphylococcal enterotoxins SEA (*sea*), SEB (*seb*), SEC (*sec*), SED (*sed*), SEE (*see*), SEG (*seg*), SEH (*seh*), SEI (*sei*), SEJ (*sej*); exfoliative toxin A, B (ETA; *eta*, ETB; *etb*, respectively); toxic shock syndrome toxin (TSST-1; *tst*); and Panton-Valentine leukocidin (PVL; *lukS* and *lukF*) were detected by PCRs as reported previously^70–72^. Using 5 μL of PCR sample, DNA fragments were analyzed by electrophoresis on a 1% agarose gel stained with ethidium bromide.

### WGS and additional molecular phylogenetic analysis

The Nextera XT DNA sample preparation kit (Illumina Inc., San Diego, CA, USA) was used for sample preparation for WGS. The DNA libraries were then purified using AMPure beads (Beckman Coulter, Inc., CA), according to the manufacturer’s protocol. Sequencing was performed using a paired-end 2 × 250 or 300-bp cycle runs on the Illumina MiSeq sequencing system using MiSeq reagent kit v2 or v3 (Illumina Inc.).

After sequencing, the obtained reads were filtered and trimmed by removing bases with quality value scores of 20 or less, de novo assembly was performed using the CLC Genomics Workbench version 9 (Qiagen N.V., Venlo, The Netherlands) with the default parameters.

Assembled contigs were submitted to spaTyper 1.0 for *spa*-typing, ResFinder 3.0 for detection of acquired drug-resistant genes, and MLST 1.8 for MLST, which are all housed at the Center for Genomic Epidemiology (CGE) website (http://www.genomicepidemiology.org//)^16,73–75^.

To infer the phylogenetic relationship based on whole-genome SNPs among strains in this study and 125 reference strains, assembled contigs were also submitted to CSI phylogeny 1.4 on the CGE website^76^. The complete sequences of the reference strains were accessed from the National Center for Biochemistry Information (NCBI) database. In addition, pairwise SNP analyses were performed focusing on CC5 and CC30 strains in order to elucidate relatedness with and preservation among the recent MRSA strains. Using a Newick file output from SNP analysis by CSI phylogeny, a neighbor-joining (NJ) tree was visualized using Figtree v1.4.3 (http://tree.bio.ed.ac.uk/software/figtree/). The numbers of inter-strain SNP differences were constructed in a red-yellow-green gradient with red indicating the top score (> 600) and green indicating the bottom score (0).

## Data Availability

The read data for whole-genome sequencing analysis of strains in this study have been deposited in GenBank under accession number DRA010146.

## References

[1] Kluytmans J, van Belkum A, Verbrugh H (1997). Nasal carriage of *Staphylococcus aureus*: Epidemiology, underlying mechanisms, and associated risks. Clin. Microbiol. Rev..

[2] Jevons MP, Coe AW, Parker MT (1963). Methicillin resistance in staphylococci. Lancet.

[3] Barber M (1961). Methicillin-resistant staphylococci. J. Clin. Pathol..

[4] Enright MC (2002). The evolutionary history of methicillin-resistant *Staphylococcus aureus* (MRSA). Proc. Natl. Acad. Sci. USA.

[5] Gorak EJ, Yamada SM, Brown JD (1999). Community-acquired methicillin-resistant *Staphylococcus aureus* in hospitalized adults and children without known risk factors. Clin. Infect. Dis..

[6] Hiramatsu K (2013). Genomic basis for methicillin resistance in *Staphylococcus aureus*. Infect. Chemother..

[7] Boucher HW, Corey GR (2008). Epidemiology of methicillin-resistant *Staphylococcus aureus*. Clin. Infect. Dis..

[8] Ito T, Katayama Y, Hiramatsu K (1999). Cloning and nucleotide sequence determination of the entire *mec* DNA of pre-methicillin-resistant *Staphylococcus aureus* N315. Antimicrob. Agents Chemother..

[9] Wu Z, Li F, Liu D, Xue H, Zhao X (2015). Novel Type XII Staphylococcal Cassette Chromosome *mec* Harboring a New Cassette Chromosome Recombinase, CcrC2. Antimicrob. Agents Chemother..

[10] Baig S (2018). Novel SCC*mec* type XIII (9A) identified in an ST152 methicillin-resistant *Staphylococcus aureus*. Infect. Genet. Evol..

[11] Urushibara N, Aung MS, Kawaguchiya M, Kobayashi N (2020). Novel staphylococcal cassette chromosome *mec* (SCC*mec*) type XIV (5A) and a truncated SCC*mec* element in SCC composite islands carrying *speG* in ST5 MRSA in Japan. J. Antimicrob. Chemother..

[12] Hiramatsu K, Kondo N, Ito T (1996). Genetic basis for molecular epidemiology of MRSA. J. Infect. Chemother..

[13] Hiramatsu K, Cui L, Kuroda M, Ito T (2001). The emergence and evolution of methicillin-resistant *Staphylococcus aureus*. Trends Microbiol..

[14] Kondo Y (2007). Combination of multiplex PCRs for staphylococcal cassette chromosome *mec* type assignment: rapid identification system for *mec*, *ccr*, and major differences in junkyard regions. Antimicrob. Agents Chemother..

[15] Oliveira DC, de Lencastre H (2002). Multiplex PCR strategy for rapid identification of structural types and variants of the *mec* element in methicillin-resistant *Staphylococcus aureus*. Antimicrob. Agents Chemother..

[16] Larsen MV (2012). Multilocus sequence typing of total-genome-sequenced bacteria. J. Clin. Microbiol..

[17] Nielsen KL (2012). Fitness cost: A bacteriological explanation for the demise of the first international methicillin-resistant *Staphylococcus aureus* epidemic. J. Antimicrob. Chemother..

[18] Campanile F, Bongiorno D, Borbone S, Stefani S (2009). Hospital-associated methicillin-resistant *Staphylococcus aureus* (HA-MRSA) in Italy. Ann. Clin. Microbiol. Antimicrob..

[19] Li S (2018). The changing pattern of population structure of *Staphylococcus aureus* from Bacteremia in China from 2013 to 2016: ST239-030-MRSA Replaced by ST59-t437. Front. Microbiol..

[20] McGavin MJ, Arsic B, Nickerson NN (2012). Evolutionary blueprint for host- and niche-adaptation in *Staphylococcus aureus* clonal complex CC30. Front Cell Infect. Microbiol..

[21] Tewhey R (2012). Genetic structure of community acquired methicillin-resistant *Staphylococcus aureus* USA300. BMC Genomics.

[22] Alam, M. T. *et al.* Transmission and microevolution of USA300 MRSA in U.S. households: Evidence from whole-genome sequencing. *mBio* **6**, e00054–00015. 10.1128/mBio.00054-15 (2015). 10.1128/mBio.00054-15 PMC4453535 25759497

[23] Vogel V, Falquet L, Calderon-Copete SP, Basset P, Blanc DS (2012). Short term evolution of a highly transmissible methicillin-resistant *Staphylococcus aureus* clone (ST228) in a tertiary care hospital. PLoS ONE.

[24] Kuroda M, Kuwahara-Arai K, Hiramatsu K (2000). Identification of the up- and down-regulated genes in vancomycin-resistant *Staphylococcus aureus* strains Mu3 and Mu50 by cDNA differential hybridization method. Biochem. Biophys. Res. Commun..

[25] Taneike I (2006). Molecular nature of methicillin-resistant *Staphylococcus aureus* derived from explosive nosocomial outbreaks of the 1980s in Japan. FEBS Lett..

[26] Gillespie WA, Alder VG (1957). Control of an outbreak of staphylococcal infection in a hospital. Lancet.

[27] Rountree PM, Rheuben J (1956). Penicillin-resistant Staphylococci in the general population. Med. J. Aust..

[28] Rountree PM, Beard MA (1958). Further observations on infection with phage type 80 staphylococci in Australia. Med. J. Aust..

[29] Shaffer, T. E., Sylvester, R. F., Jr., Baldwin, J. N. & Rheins, M. S. Staphylococcal infections in newborn infants. II. Report of 19 epidemics caused by an identical strain of staphylococcus pyogenes. *Am. J. Public Health Nations Health* **47**, 990–994. 10.2105/ajph.47.8.990 (1957). 10.2105/ajph.47.8.990 PMC1551268 13444510

[30] Bynoe ET, Elder RH, Comtois RD (1956). Phage-typing and antibiotic-resistance of staphylococci isolated in a general hospital. Can. J. Microbiol..

[31] Jessen O, Rosendal K, Bülow P, Faber V, Eriksen KR (1969). Changing Staphylococci and Staphylococcal Infections. N. Engl. J. Med..

[32] Rodriguez-Noriega E (2010). Evolution of methicillin-resistant *Staphylococcus aureus* clones in Latin America. Int. J. Infect. Dis..

[33] Aires De Sousa, M. *et al.* Three-year assessment of methicillin-resistant *Staphylococcus aureus* clones in Latin America from 1996 to 1998. *J. Clin. Microbiol.* **39**, 2197–2205. 10.1128/JCM.39.6.2197-2205.2001 (2001). 10.1128/JCM.39.6.2197-2205.2001 PMC88111 11376057

[34] Sola C, Cortes P, Saka HA, Vindel A, Bocco JL (2006). Evolution and molecular characterization of methicillin-resistant *Staphylococcus aureus* epidemic and sporadic clones in Cordoba Argentina. J. Clin. Microbiol..

[35] Becker AP, Santos O, Castrucci FM, Dias C, D'Azevedo PA (2012). First report of methicillin-resistant *Staphylococcus aureus* Cordobes/Chilean clone involved in nosocomial infections in Brazil. Epidemiol. Infect..

[36] Teixeira MM (2012). Emergence of clonal complex 5 (CC5) methicillin-resistant *Staphylococcus aureus* (MRSA) isolates susceptible to trimethoprim-sulfamethoxazole in a Brazilian hospital. Braz. J. Med. Biol. Res..

[37] Kuroda M (2001). Whole genome sequencing of meticillin-resistant *Staphylococcus aureus*. Lancet.

[38] Imai D. Characteristics of MRSA strains isolated in Japan in 1999. *Juntendo Med. J.* **49**. 10.14789/pjmj.49.343 (2003).

[39] Ito T (2001). Structural comparison of three types of staphylococcal cassette chromosome *mec* integrated in the chromosome in methicillin-resistant *Staphylococcus aureus*. Antimicrob. Agents Chemother..

[40] Aiba Y (2013). Mutation of RNA polymerase beta-subunit gene promotes heterogeneous-to-homogeneous conversion of beta-lactam resistance in methicillin-resistant *Staphylococcus aureus*. Antimicrob. Agents Chemother..

[41] Kondo N, Kuwahara-Arai K, Kuroda-Murakami H, Tateda-Suzuki E, Hiramatsu K (2001). Eagle-type methicillin resistance: New phenotype of high methicillin resistance under *mec* regulator gene control. Antimicrob. Agents Chemother..

[42] Kuroda M (2003). Two-component system VraSR positively modulates the regulation of cell-wall biosynthesis pathway in *Staphylococcus aureus*. Mol. Microbiol..

[43] Yamakawa J (2012). Heterogeneously vancomycin-intermediate *Staphylococcus aureus* (hVISA) emerged before the clinical introduction of vancomycin in Japan: A retrospective study. J. Infect. Chemother..

[44] Harada D (2018). Change in genotype of methicillin-resistant *Staphylococcus aureus* (MRSA) affects the antibiogram of hospital-acquired MRSA. J. Infect. Chemother..

[45] Dominguez MA, de Lencastre H, Linares J, Tomasz A (1994). Spread and maintenance of a dominant methicillin-resistant *Staphylococcus aureus* (MRSA) clone during an outbreak of MRSA disease in a Spanish hospital. J. Clin. Microbiol..

[46] Mato R (1998). Spread of the multiresistant Iberian clone of methicillin-resistant *Staphylococcus aureus* (MRSA) to Italy and Scotland. Microb. Drug Resist..

[47] Roberts RB (1998). Outbreak in a New York City teaching hospital burn center caused by the Iberian epidemic clone of MRSA. Microb. Drug Resist..

[48] Sanches IS (1995). Evidence for the geographic spread of a methicillin-resistant *Staphylococcus aureus* clone between Portugal and Spain. J. Clin. Microbiol..

[49] Lakhundi, S. & Zhang, K. Methicillin-Resistant *Staphylococcus aureus*: Molecular characterization, evolution, and epidemiology. *Clin. Microbiol. Rev.* **31**. 10.1128/CMR.00020-18 (2018). 10.1128/CMR.00020-18 PMC6148192 30209034

[50] Hryniewicz MM, Garbacz K (2017). Borderline oxacillin-resistant *Staphylococcus aureus* (BORSA): A more common problem than expected?. J. Med. Microbiol..

[51] Goering RV, Swartzendruber EA, Obradovich AE, Tickler IA, Tenover FC (2019). Emergence of oxacillin resistance in stealth methicillin-resistant *Staphylococcus aureus* due to *mecA* sequence instability. Antimicrob. Agents Chemother..

[52] Nelson L (2006). Community case of methicillin-resistant *Staphylococcus aureus* Infection. Emerg. Infect. Dis..

[53] McDougal LK, Thornsberry C (1986). The role of beta-lactamase in staphylococcal resistance to penicillinase-resistant penicillins and cephalosporins. J. Clin. Microbiol..

[54] Tomasz A (1989). New mechanism for methicillin resistance in *Staphylococcus aureus*: clinical isolates that lack the PBP 2a gene and contain normal penicillin-binding proteins with modified penicillin-binding capacity. Antimicrob. Agents Chemother..

[55] Yoshida R (2003). Physiological and molecular analysis of a *mecA*-negative *Staphylococcus aureus* clinical strain that expresses heterogeneous methicillin resistance. J. Antimicrob. Chemother..

[56] Hackbarth CJ, Kocagoz T, Kocagoz S, Chambers HF (1995). Point mutations in *Staphylococcus aureus* PBP 2 gene affect penicillin-binding kinetics and are associated with resistance. Antimicrob. Agents Chemother..

[57] Nadarajah J (2006). Identification of different clonal complexes and diverse amino acid substitutions in penicillin-binding protein 2 (PBP2) associated with borderline oxacillin resistance in Canadian *Staphylococcus aureus* isolates. J. Med. Microbiol..

[58] Berger-Bachi B, Rohrer S (2002). Factors influencing methicillin resistance in staphylococci. Arch. Microbiol..

[59] Roemer T, Schneider T, Pinho MG (2013). Auxiliary factors: a chink in the armor of MRSA resistance to beta-lactam antibiotics. Curr. Opin. Microbiol..

[60] Ba X (2019). Truncation of GdpP mediates beta-lactam resistance in clinical isolates of *Staphylococcus aureus*. J. Antimicrob. Chemother..

[61] Banerjee R, Gretes M, Harlem C, Basuino L, Chambers HF (2010). A *mecA*-negative strain of methicillin-resistant *Staphylococcus aureus* with high-level beta-lactam resistance contains mutations in three genes. Antimicrob. Agents Chemother..

[62] Argudin, M. A. *et al.* Genetic diversity among *Staphylococcus aureus* isolates showing oxacillin and/or cefoxitin resistance not linked to the presence of *mec* genes. *Antimicrob. Agents Chemother.* **62**. 10.1128/aac.00091-18 (2018). 10.1128/AAC.00091-18 PMC6021684 29661881

[63] Matsumoto K *et al.* The pathogenic strains of *Staphylococcus aureus* lately isolated in Japan. *Chemotherapy* **32**. 10.11250/chemotherapy1953.32.527 (1984).

[64] Sasaki T (2010). Multiplex-PCR method for species identification of coagulase-positive staphylococci. J. Clin. Microbiol..

[65] Hiramatsu K, Kihara H, Yokota T (1992). Analysis of borderline-resistant strains of methicillin-resistant *Staphylococcus aureus* using polymerase chain reaction. Microbiol. Immunol..

[66] Murakami K (1991). Identification of methicillin-resistant strains of staphylococci by polymerase chain reaction. J. Clin. Microbiol..

[67] Enright MC, Day NP, Davies CE, Peacock SJ, Spratt BG (2000). Multilocus sequence typing for characterization of methicillin-resistant and methicillin-susceptible clones of *Staphylococcus aureus*. J. Clin. Microbiol..

[68] Harmsen D (2003). Typing of methicillin-resistant *Staphylococcus aureus* in a university hospital setting by using novel software for *spa* repeat determination and database management. J. Clin. Microbiol..

[69] Strommenger B (2008). *spa* Typing of *Staphylococcus aureus* as a frontline tool in epidemiological typing. J. Clin. Microbiol..

[70] Becker K, Roth R, Peters G (1998). Rapid and specific detection of toxigenic *Staphylococcus aureus*: use of two multiplex PCR enzyme immunoassays for amplification and hybridization of staphylococcal enterotoxin genes, exfoliative toxin genes, and toxic shock syndrome toxin 1 gene. J. Clin. Microbiol..

[71] Lina G (1999). Involvement of Panton-Valentine leukocidin-producing *Staphylococcus aureus* in primary skin infections and pneumonia. Clin. Infect. Dis..

[72] Monday SR, Bohach GA (1999). Use of multiplex PCR to detect classical and newly described pyrogenic toxin genes in staphylococcal isolates. J. Clin. Microbiol..

[73] Joensen KG (2014). Real-time whole-genome sequencing for routine typing, surveillance, and outbreak detection of verotoxigenic *Escherichia coli*. J. Clin. Microbiol..

[74] Zankari E (2012). Identification of acquired antimicrobial resistance genes. J. Antimicrob. Chemother..

[75] Bartels MD (2014). Comparing whole-genome sequencing with Sanger sequencing for *spa* typing of methicillin-resistant *Staphylococcus aureus*. J. Clin. Microbiol..

[76] Kaas RS, Leekitcharoenphon P, Aarestrup FM, Lund O (2014). Solving the problem of comparing whole bacterial genomes across different sequencing platforms. PLoS ONE.

